# Editorial: Application of nanotechnology in diagnosis and/or therapy of non-small cell lung cancer

**DOI:** 10.3389/fonc.2023.1234727

**Published:** 2023-06-16

**Authors:** Qinglin Shen, Ling Peng, Yi Zhang, Ruoxiang Wang

**Affiliations:** ^1^ Department of Oncology, Jiangxi Provincial People’s Hospital, the First Affiliated Hospital of Nanchang Medical College, Nanchang, China; ^2^ Institute of Clinical Medicine, Jiangxi Provincial People’s Hospital, the First Affiliated Hospital of Nanchang Medical College, Nanchang, China; ^3^ Department of Respiratory Disease, Zhejiang Provincial People’s Hospital, Affiliated People’s Hospital, Hangzhou Medical College, Hangzhou, China; ^4^ Department of Biomedical Science, Samuel Oschin Comprehensive Cancer Center, Cedars-Sinai Medical Center, Los Angeles, CA, United States; ^5^ Department of Medicine, Samuel Oschin Comprehensive Cancer Center, Cedars-Sinai Medical Center, Los Angeles, CA, United States

**Keywords:** nanomaterials (A), cancer diagnosis, cancer therapy, precision medicine, non-small cell lung cancer (NSCLC)

Lung cancer is one of the malignant tumors with the fastest growth in incidence and mortality and the greatest threat to human health and life. Non-small cell lung cancer (NSCLC) accounts for approximately 80% of all lung cancers, with approximately 75% of patients diagnosed in the advanced stage, and the 5-year survival rate of advanced patients is less than 5% (1). Important advances in the diagnosis and therapy of NSCLC have been achieved over the past decades. Targeted therapy and immunotherapy bring significant survival benefits to the selected NSCLC patients, but the overall cure and survival rates for NSCLC are still unsatisfactory, particularly in advanced patients. The development of nanotechnology has brought new hope for the precise diagnosis and treatment of cancer.

Compared with previous diagnostic methods, nanotechnology has advantages such as small size, good biocompatibility, and strong organ targeting ability, providing a new multifunctional platform for biomedical research (2). Nanotechnology has broad application prospects in the diagnosis of tumors, especially in nuclear magnetic resonance imaging (MRI) technology. Nanoscale nuclear MRI technology significantly improves the precision and accuracy of tumor diagnosis. A lot of energy is devoted to the development of precise diagnosis to achieve early diagnosis for NSCLC. Endobronchial optical coherence tomography (EB-OCT) has the characteristics of non-invasiveness, accuracy, and repeatability. Importantly, the combination of EB-OCT with existing technologies represents a potential approach for early screening and diagnosis. EB-OCT is of great significance in the diagnosis of lung cancer and suitable for clinical application and is expected to become an important diagnostic method for lung cancer in the future (Long et al.).

Nanotechnology has emerged as a modality for the treatment of cancer. Combination therapies with high efficacy and low toxicity are required to extend the clinical benefit to a wider patient population and improve the prognosis of NSCLC (3). Functional biomaterials designed by nanotechnology can be used for targeted drug delivery, which can deliver drugs to tumor sites at fixed points, thereby reducing the toxic and side effects of drugs on normal tissues. At the same time, nanomaterials constructed by nanotechnology can significantly enhance the sensitivity and resolution of diagnosis, providing the possibility for early diagnosis and timely treatment of tumors. Peng et al. believed that with the novel engineering avenues of nanomedicine, there is a possibility to overcome the inherent limitations derived from conventional and emerging treatments, such as off-site drug cytotoxicity, drug resistance, and administration methods. Combining nanotechnology with the convergence points of current therapies could open up new avenues for meeting the unmet needs of NSCLC treatment.

Nanocarriers can greatly improve the dosage and accuracy of targeted drug release, as well as reduce toxicity and side effects, thus treating tumors more effectively in a non-invasive state in the human body. In combination with nanoparticles, it is possible to improve the pharmacokinetic and pharmacodynamic profiles of pre-existing drugs used in cancer treatment. Nanoparticles have physiochemical properties such as small size, allowing passage through challenging areas of the body, and a large surface area allows for higher doses of drugs to be brought to the tumor site. Nanoparticles can be functionalized, which involves modifying the surface chemistry of the particles and allows for the conjugation of ligands (small molecules, antibodies, and peptides). Ligands can be chosen for their ability to target components that are specific to or are upregulated in cancer cells, such as targeting receptors on the tumor surface that are highly expressed in the cancer. This ability to precisely target the tumor can improve the efficacy of drugs and decrease toxic side effects. This review will discuss approaches used for targeting drugs to tumors using nanoparticles, provide examples of how this has been applied in the clinic, and highlight future prospects for this technology (Holder et al.).

Sun et al. review the advances in research on antitumor immunomodulation in Chinese herbal medicine, including the regulation of the innate immune system, which includes macrophages, MDSCs, and natural killer cells, and the adaptive immune system, which includes CD4+ T cells, CD8+ T cells, and regulatory T cells (Tregs), to influence tumor-associated inflammation. In addition, a combination of active ingredients of herbal medicine and modern nanotechnology alters the tumor immune microenvironment. In recent years, immunological antitumor therapy in TCM has been applied on a reasonably large scale both nationally and internationally, and there is potential for further clinical expansion. Investigation of immune modulation mechanisms in Chinese herbal medicine will provide novel perspectives of how herbal medicine controls tumor growth and metastasis, which will contribute to the evolution of tumor research.

Therefore, we expect nanotechnology to bring precise diagnosis and treatment to patients with NSCLC, improving their overall survival rate and quality of life.

## Author contributions

QS drafted the manuscript; QS, LP, YZ, and RW reviewed and edited the manuscript. All authors contributed to the article and approved the submitted version.
